# Advances in cancer mechanobiology: Metastasis, mechanics, and materials

**DOI:** 10.1063/5.0186042

**Published:** 2024-03-05

**Authors:** Abigail J. Clevenger, Maygan K. McFarlin, John Paul M. Gorley, Spencer C. Solberg, Anirudh K. Madyastha, Shreya A. Raghavan

**Affiliations:** 1 Department of Biomedical Engineering, Texas A&M University, College Station, Texas 77843, USA; 2 CPRIT Regional Center of Excellence in Cancer, Texas A&M University, College Station Texas 77843, USA

## Abstract

Within the tumor microenvironment (TME), tumor cells are exposed to numerous mechanical forces, both internally and externally, which contribute to the metastatic cascade. From the initial growth of the tumor to traveling through the vasculature and to the eventual colonization of distant organs, tumor cells are continuously interacting with their surroundings through physical contact and mechanical force application. The mechanical forces found in the TME can be simplified into three main categories: (i) shear stress, (ii) tension and strain, and (iii) solid stress and compression. Each force type can independently impact tumor growth and progression. Here, we review recent bioengineering strategies, which have been employed to establish the connection between mechanical forces and tumor progression. While many cancers are explored in this review, we place great emphasis on cancers that are understudied in their response to mechanical forces, such as ovarian and colorectal cancers. We discuss the major steps of metastatic transformation and present novel, recent advances in model systems used to study how mechanical forces impact the study of the metastatic cascade. We end by summarizing systems that incorporate multiple forces to expand the complexity of our understanding of how tumor cells sense and respond to mechanical forces in their environment. Future studies would also benefit from the inclusion of time or the aspect of mechanical memory to further enhance this field. While the knowledge of mechanical forces and tumor metastasis grows, developing novel materials and *in vitro* systems are essential to providing new insight into predicting, treating, and preventing cancer progression and metastasis.

## I. INTRODUCTION: THE MECHANICS OF METASTASIS

**FIG. 1.**
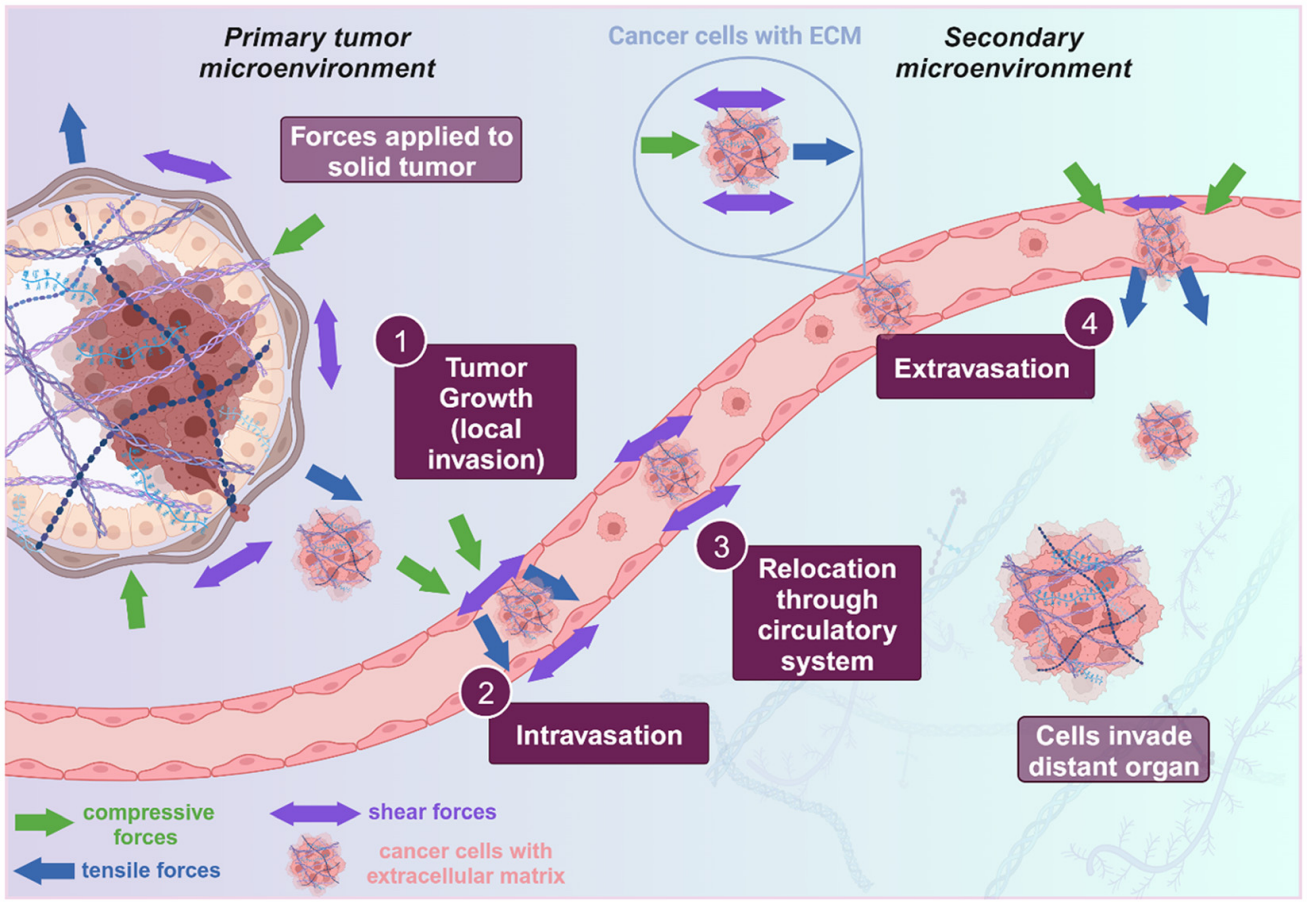
Metastatic cascade: The metastatic cascade is comprised of four major steps: (i) tumor growth or local invasion into surrounding tissue, (ii) intravasation of tumor cells into the circulatory or lymphatic systems, (iii) relocation of tumor cells through circulation, and (iv) extravasation of tumor cells out of vasculature and into distant organ sites. Throughout the metastatic cascade, numerous forces are at play, including compressive, tensile, and shear forces, as indicated by the colored arrows. The figure was created on Biorender.

The process of metastasis has long been studied to understand how cells escape from confined tumor spaces to become free floating cell clusters that colonize distant organs.^1–4^ This entire process is defined as the metastatic cascade and, in general, hematogenous metastasis is composed of four major steps: (i) local invasion into surrounding tissue, (ii) intravasation into blood and lymphatic vasculature, (iii) relocation through the circulatory system, and (iv) extravasation from vascular tissues into distant organs (Fig. 1).^5,6^

From the initial state as a solid tumor, through the shift to metastatic, free-floating cell clusters, and eventually to the colonization of a distant organ, the presence of mechanical forces is ubiquitous and integral to the progression of cancer (Fig. 1).^7^ Mechanical forces are transduced through mechanoreceptors which then affect cellular processes, such as proliferation and differentiation, well reviewed by Bera *et al.*^8^ Specifically, mechanical cues alter cell morphology, cell–cell interaction, and cell signaling and facilitate epithelial to mesenchymal transition (EMT) and other migratory programming.^9,10^ Forces within the tumor microenvironment (TME) can be broken down and simplified into three categories: (i) shear stress, (ii) tension and strain, and (iii) solid stress and compression (Fig. 1). At the tumor cell level, forces experienced are typically nuanced combinations of these mechanical stressors. This review breaks down each category of forces individually to focus on recent, specific models to understand how mechanical forces impact tumor progression and metastasis. In breaking down complicated forces into modular individual mechanical stimuli, the review also highlights how some cancers are disproportionately studied for the influence of mechanical forces on metastasis, while some others are significantly understudied.

During the metastatic cascade, each force type plays an explicit role in the progression of cancer. In a biological context, fluid shear stress is the force exerted by fluid flow through a system and is prominent throughout the body, most obviously in the vasculature. Therefore, fluid shear stress is predominant in the circulation of cancer cells as they migrate to colonize distant organs.^11–17^ Shear stress is also experienced by stationary tumors where fluid flow is prevalent in the microenvironment, such as in colorectal, endometrial, bladder, and ovarian cancers.^18–22^ Mechanical strain, tension, solid stress, and compression also have more targeted effects on tumors as they are more localized forces. Strain is often discussed physiologically as an effect of tensile forces or stretching forces in the body, both of which are markers and causes of tumorigenesis and metastasis.^23–26^ Strain caused by tensile forces factors into nearly any tumor microenvironment (TME) through the extracellular matrix (ECM), which relays mechanical force from external sources, or endogenous tumor growth, to individual cells.^27^ Substrate stiffness plays an important role in tensile forces experienced by tumors as increased stiffness increases tension in the tumor's ECM.^28,29^ Furthermore, the cancer TME reprogramming of the ECM alters substrate stiffness leading to a vicious cycle of aberrant stiffness in both the tumor and the microenvironment. Strain is also experienced in the form of mechanical stretching in cancers, such as lung and colorectal, due to the inherent cyclic motion occurring at the tissue and organ levels.^26,30^ Solid stress is a force that comes from the compression of an object's interior due to the growth and expansion of the object itself. In the cancer microenvironment, solid stress due to unmitigated tumor growth results in the compression of cells on the interior of the tumor. Solid stress can also manifest as compression experienced by elevated interstitial pressure from unregulated tumor growth. Compression and solid stress encourage migration and metastasis of cancer cells to alleviate the increased pressure in the TME. Clearly each force type is important to the progression and metastasis of cancer cells, and they all uniquely interact with the TME throughout the metastatic cascade. Therefore, the development of integrative biomaterial and tissue models are essential to accurately mimic these forces in the *in vitro* study of metastasis mechanobiology.

Traditionally, mechanobiology has relied heavily on 2D model systems with applied physical stimuli.^31^ This methodology was instrumental in the early understanding of mechanotransduction and the response of cancer cells to mechanical forces resulting in invasion and metastasis. However, the TME is a complex 3D entity, existing with a 3D spatial organization. The investment and growth of 3D *in vitro* model systems have shown significant differences in biological aspects of cells in 2D vs 3D microenvironments, prompting the shift toward 3D systems in the study of cancer mechanobiology.^32–34^ Several tissue engineering and regenerative medicine strategies have even been adapted to model cancer processes.^35^ With the integration of mechanical forces, these newer model systems essentially incorporate the 3D element of cancer mechanobiology, thereby allowing researchers to better pinpoint specific force interactions that contribute to metastatic progression.

In this review, we discuss novel recent models used to study how mechanical forces play a role in the steps of the metastatic cascade. We break down each force type individually and include model systems used to study shear stress, tension and strain, and solid stress and compression in the space of cancer progression and metastasis. While many types of cancer are included in this work, we paid special attention to traditionally understudied cancers (especially as they pertain to mechanobiology), such as ovarian and colorectal, relative to their incidence (Table I).^36^ We close this work by briefly describing systems that incorporate multiple force types to increase the complexity of our understanding of mechanics and metastasis. This review highlights many recent and innovative materials and *in vitro* systems that are essential to developing methods for predicting, treating, and preventing cancer progression and metastasis.

**TABLE I.** Summary of mechanical forces applied on ovarian and colorectal cancer cells including cell type, force applied, force magnitude, and observed cell response.

Cancer	Cell type	Force applied	Magnitude	Cell response	Reference
Ovarian cancer	SKOV-3	Fluid shear applied in both horizontal and vertical directions via lab line rotator	0.13–0.32 dyne/cm^2^	Increase in capacity for spheroid formation, disorganization of actin stress fibers, development of actin-containing protrusions	Hyler *et al.*^19^
	OVCAR-3	Wall shear stress applied on cell surface	0.5, 1.0, 1.5 dyne/cm^2^	Cell elongation, stress fiber formation, and microtubule generation	Avraham-Chakim *et al.*^100^
	SKOV-3	Tension forces applied to separate membrane from cytoskeleton	190 ± 79 pN	Cancerous cells required significantly less force than normal epithelial cancer cells showing an increases susceptibility to mechanical deformation	Lu *et al.*^101^
	SKOV-3 OVCAR-8	Oscillatory tension for three days	10% elongation at 0.3 Hz	Increased cell proliferation and expression of proliferation genes (FOXS1 and ERVV-2)	Martinez *et al.*^88^
	OVCAR3 OVSAHO	Cyclic and static compression forces applied for 24 and 72 h	52 000 dyne/cm^2^ static 39 000–65 000 dyne/cm^2^ cyclic	Increased invasive morphology, proliferation, and chemoresistance via CDC42	Novak *et al.*^97^
	OVCA429 OVCA433 DOV13 SKOV-3	Compression forces applied for 6 h using FlexCell	25 mmHg	Decrease in EMT related gene expression	Klymenko *et al.*^95^
	OVCA429 OVCA433 DOV13 SKOV-3	Compression forces applied for 24 h using FlexCell	25 mmHg	Increased expression of EMT related genes	Klymenko *et al.*^95^
Colorectal cancer	LoVo SW480 SW620 DLD1 HT29 HCT116	Laminar shear stress	5 dyne/cm^2^ 10 dyne/cm^2^ 20 dyne/cm^2^	Increased ATOH8 expression (suppression of cell death)	Huang *et al.*^44^
	cancer tissue-originated spheroids	Hydrodynamic stress via syringe pump	Passed through syringe at ∼15 ml/min	Increased growth of cancer cell clusters	Hagihara *et al.*^102^
	SW480 HCT116	Laminar shear stress	15 dyne/cm^2^ for 12 h	P38 phosphorylation increased, expression of β-catenin target genes decreased	Avvisato *et al.*^103^
	HCT116 PDX1,2 HIEC-6	Shear and cyclic strain	4 dyne/cm^2^ shear 15% cyclic strain	Increase in LGR5+ cells, epithelial to mesenchymal plasticity gene expression, and invasive morphology changes	Clevenger *et al.*^104^
	SW480	Oscillatory shear strain	90 Hz for 4 h	Increased apoptosis in colorectal cancer cells	Garteiser *et al.*^105^

## II. BIOENGINEERING STRATEGIES TO STUDY CANCER CELL RESPONSE TO SHEAR STRESS

Fluid shear in blood and lymphatic vessels, as well as interstitial spaces, is primarily known to facilitate metastasis by promoting the circulation of the many solid and liquid cancers that spread through these systems.^11–13,15–17^ Variations in fluid shear in the tumor microenvironment also have elicit a direct cellular response leading to the progression and metastasis of breast, ovarian, and colorectal cancers shown through *in vitro* studies^18–20^ (Table I). Along with the general transport of cancer cells, *in vitro* studies where cancer cells were exposed to fluid shear stress have shown specific induction of genomic instability^19^ and genetic reprogramming into stem-cell like states to survive new mechanical microenvironments.^37^ Fluid shear stress also results in changes in cellular morphology portending invasiveness,^19^ changes in proliferative ability,^20^ and activation of mechanosensors like YAP1 to increase cellular motility and alter the cell cycle.^38,39^ As knowledge of the relationship between shear stress and metastasis has grown, the need for innovative, *in vitro* strategies to model the interplay of shear force and cancer cells has grown in tandem.

**FIG. 2.**
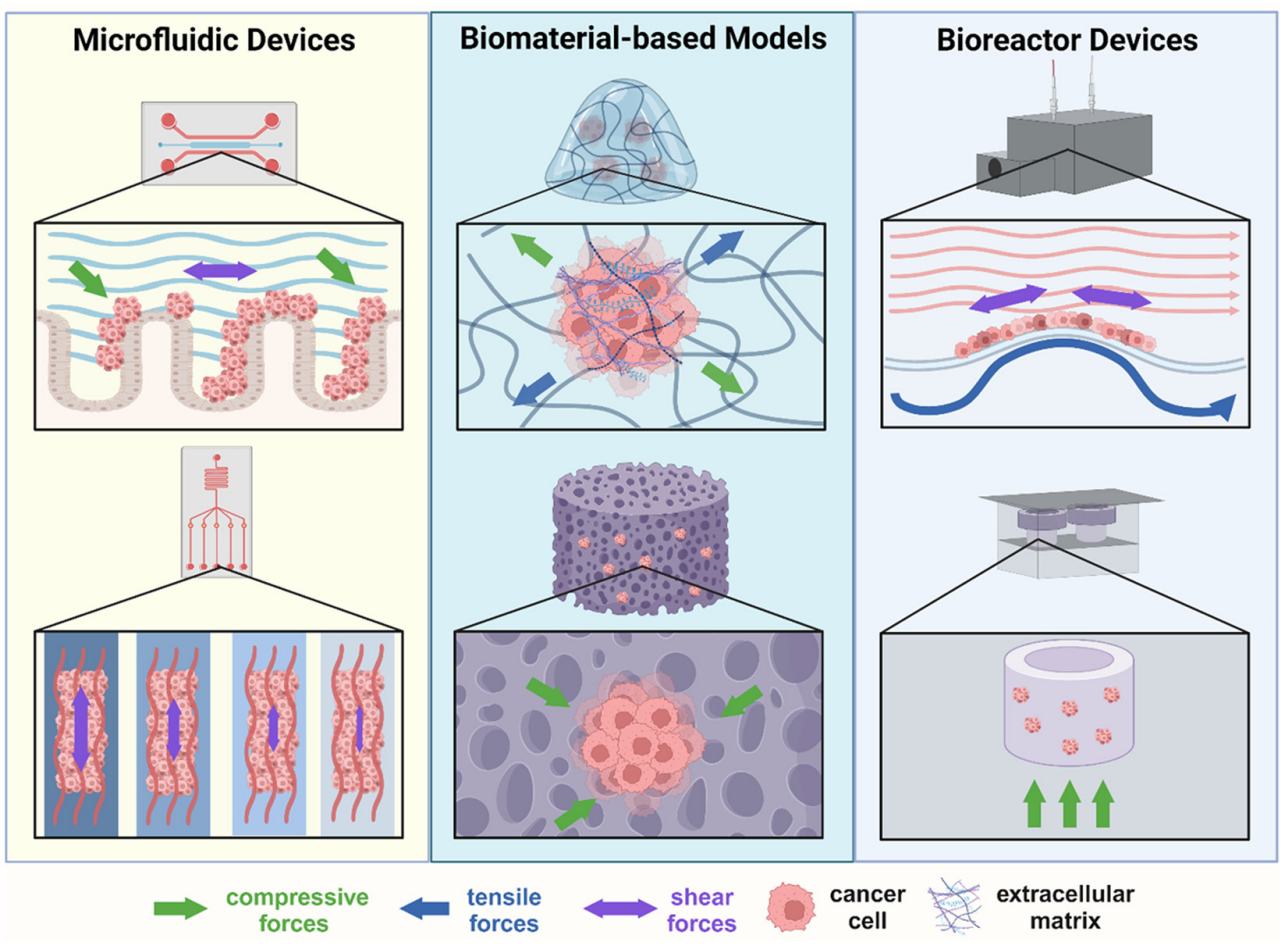
Biongineering strategies: Illustrations of various models and devices employed to evaluate the impact of mechanical forces on cancer cells. Microfluidic devices, biomaterial-based models, and bioreactor devices are all utilized to study force interactions, indicated by the colored arrows, with cancer cells. The figure is created on Biorender.

A rapidly emerging technique for studying the interactions between mechanical forces and cancer is organ-on-a-chip technology. Through microfluidic channels, exact spatial organization, minute mechanical force adjustments, and cell–cell or tissue–tissue interactions can be fine-tuned to closely match conditions in the body.^40^ Exploring shear-induced tumor progression using organ-on-a-chip, Strelez *et al.* developed a colorectal cancer microchip for comparison with a healthy intestine-on-a-chip model. They found decreased epithelial cadherin and increased vimentin, as well as increased invasion, in the colorectal cancer chip model with fluid shear stress.^41^ By incorporating mechanical force, endothelial interactions, and the addition of fibroblasts, this innovative model system recapitulated numerous factors present in the *in vivo* environment. The required complexity of microengineering needed to induce organ-like behavior *in vitro* is certainly an important consideration in making organ-on-a-chip devices universally accessible for *in vitro* study. Looking at a more simplified model, Dash *et al.* evaluated HeLa cervical cancer cells in a microfluidic device specifically designed to efficiently compare four levels of shear stress in one experiment. This work found a correlation between increasing shear stress magnitude [0.01–10 dyne/cm^2^] and enhancement of stemness-related genes Sox2 and N-cadherin.^42^ The up-regulation of N-cadherin, in particular, is also interesting because it indicates a motile mesenchymal genotype.^43^ Similarly, in a study on colorectal cancer cells, the application of multiple values of laminar shear stress [0–20 dyne/cm^2^] via a microfluidic bioreactor resulted in an upregulation of ATOH8, a mechanosensor that contributes to circulating tumor cell survival (Fig. 2).^44^ In both examples, shear stress contributed to metastatic behavior in the increase in stemness-related gene expression and circulating tumor cell survival. In using simplified model systems, Dash *et al.* and Huang *et al.* looked directly at shear stress magnitude to isolate how this mechanical force alters cellular response (Table I). Magnitude of shear stresses is certainly an interesting variable to study, because it varies widely in blood flow within vessels and across organ systems with their inherent movement. Therefore, uncovering differing cell responses to magnitudes of shear highlights a survivability advantage in specific transitory microenvironments during circulatory spread (Fig. 2). In another case of the study of fluid shear force, a peristaltic pump supplied shear within a microfluidic bioreactor. SK-BR-3 breast cancer cells were circulated to mimic motion of suspended tumor cells in the blood stream. Exposure to this model produced an upregulation/downregulation of mesenchymal/epithelial markers on both gene and protein expression levels. This work highlighted the contribution of shear force in blood vessels to the EMT process in circulating tumor cells.^13^ By incorporating motion similar to that of the blood stream, this model creates a unique way to study a common route of cancer cell spread via the circulatory system. Despite this example incorporating larger scale force with the use of an external peristalsis pump, the need for small scale engineering techniques makes microfluidics less accessible to a wide audience. While microfluidics and organ-on-a-chip technology can serve their purpose, working with larger scale models introduces a more user-friendly approach to studying shear stress.

Looking at some larger scale models, in one application, fluid flow shear stress was applied to osteosarcoma cells using the commercial Flexcell fluid shear system.^45^ In this novel work on a relatively understudied cancer type, researchers determined that physiologically relevant fluid shear stress restored normal osteogenic gene expression in osteosarcoma cells. These results suggest that the modulation of fluid movement in osteosarcoma tumors could become an important consideration in the development of new treatments to prevent progression. Larger scale bioreactors, while sacrificing some control over fluidics, ultimately retain a wider breadth of application, especially when they can be tuned to mimic different biological systems.^46,47^ For example, the work by Fuh *et al.*, adapted a parallel plate flow system created to study aortic endothelial cells for the assessment of fluid shear stress on monolayers of multiple breast cancer cell lines (MDA-MB-231, SK-BR-3, BT-474, and MCF-7).^48^ Pulse dampeners were added to the system to evaluate cellular response to fluid shear stress levels found in the vasculature. The flow-exposed condition resulted in upregulation of EMT markers, Vimentin and Snail, as well as an increase in flow-stimulated MDA-MB-231 invasion through transwell membranes. The integration of genotype–phenotype changes leading from mechanical stimulus to actual cellular invasiveness was a particular advantage. This example demonstrates the versatility of larger scale systems that can be easily tuned to replicate other biological environments, such as lymphatic vessels. Another example of a large-scale model of fluid shear stress *in vitro* is the cone-and-plate viscometer system used by Mitchell *et al.* in the study of colorectal cancer cells.^49^ This work was the first of its kind to establish that physiological fluid shear force can sensitize receptor-mediated apoptosis of colorectal cancer cells in the presence of apoptotic agents. Further work with this model has incorporated the use of natural killer cells. These studies are situated at the convergence of immunology, cancer, and mechanobiology, creating a unique avenue for advanced therapeutics.^50^ The use of larger bioreactor systems addresses the issue of complexity found in microfluidics and provides an alternative to small scale shear stress studies.

While many of the strategies described above can be tuned for use in systems with prominent shear stress levels, such as the vasculature, lymphatic system, and colon, many tissues also experience shear stress due to interstitial flow. Interstitial flow is a slow fluid movement through solid tissue and provides nutrients to cells while removing metabolic waste. Importantly, as a physical transportation method and a promigratory influence on cancer cells, interstitial flow directly contributes to tumor growth and progression.^51–53^ Certain techniques described above apply to studies on interstitial flow-sourced shear stress, such as tunable microfluidic devices, which can mimic interstitial flow in the tumor microenvironment.^54^ Using a 3D invasion assay to study ERBB2/HER2-expressing breast cancer cells, Tchafa *et al.* found a link between interstitial flow shear and PI3K activation, suggesting a mechanism by which shear stress provokes tumor cell invasion and metastasis.^55,56^ However, because increased tumor size leads to higher interstitial fluid pressure,^57^ interstitial fluid impacts cancer cells not only directly through shear stress but also indirectly through increased pressure, making interstitial flow-specific models of study necessary. The increased pressure within a tumor establishes a pressure gradient, which becomes a barrier for drug delivery to the interior of the tumor and contributes to cancer cell migration.^57–60^ This barrier makes the pressure gradient component of interstitial flow a crucial piece of a mechanically accurate *in vitro* model of the tumor microenvironment. To address this need, Piotrowski-Daspit *et al.* developed a novel 3D engineered tumor model where breast cancer cells were aggregated within an ECM gel, with a hydrostatic pressure gradient varied reservoir heights and polydimethylsiloxane (PDMS) gaskets.^61,62^ In this *in vitro* model of breast cancer tumors, interstitial fluid pressure increased instances of collective migration or multicellular protrusions emanating from cellular aggregates via changes in EMT gene signatures.^63^ Interestingly, while increase in *Snail* and *Vimentin* genes are indicative of increased mesenchymal motility, the authors also saw retention of increased epithelial markers like *E-cadherin* and *Keratin-8*. Collective migration implies that a collection of cells migrates out of the original tumor, where there is still significant amounts of cellular attachment, in which case the increased retention of epithelial markers is expected.^64,65^ Increased EMT due to the increased fluid pressure, thereby promoted collective invasion. Few other systems exist at this interface of shear stress and pressure-based interstitial flow and future studies should encompass both aspects for a complete look at how these forces relate to cancer cell progression.^66^

In the implementation of these strategies for studying the role of shear stress in tumor progression and metastasis, future investigators should keep in mind the advantages of 3D culture models over their 2D counterparts. 3D culture allows for more physiologically relevant variables to be incorporated as shown by Novak *et al.* in their 3D culture-enabled shear stress bioreactor. In the 3D extracellular matrix with applied shear stress model, Novak *et al.* demonstrated that shear stress increased breast cancer invasion, proliferation, PLAU activation, and chemoresistance.^67^ By incorporating a 3D matrix, researchers were able to match the morphology and modulus of the tumor microenvironment that cannot be achieved in traditional 2D culture. Another instance of 3D culture is in the work by Rizvi *et al.* with their customizable microfluidic platform used to form 3D ovarian cancer nodules.^68^ Under the application of constant fluid flow, researchers established that flow induces epithelial-mesenchymal transition, cellular heterogeneity and biomarker modulation in 3D ovarian cancer nodules. By using a 3D platform, as opposed to 2D, researchers were able to evaluate morphological and molecular changes in a system representative of fluid dynamics in the peritoneal cavity. The intersection of 3D culture, with other methods of force application, such as bioreactors, bioprinting, and organ-on-a-chip systems, is a promising next step for *in vitro* modeling of mechanical forces like shear stress.

In further *in vitro* research on the metastatic and pro-tumoral implications of shear stress, it is important to determine whether direct tissue interaction is necessary, such as in the case of colorectal cancer where tumor cells are adherent to the colon or rectum but experience shear stress forces. Additionally, the desired level of control over fluid flow and spatial organization required and the breadth of systems the model ought to span must be established. Once these parameters are outlined, an appropriate strategy or device can be selected that fully encompasses the study of metastatic spread in response to shear stress. A basic understanding of the effects of shear stress on different cancers and in multiple biological systems first needs established through use of simpler bioreactors and microfluidic devices. Further studies should increase biological relevance by integrating additional biomimetic aspects and eventually organ-on-a-chip, especially as microtechnology continues to advance.

## III. BIOENGINEERING STRATEGIES TO STUDY CANCER CELL RESPONSE TO TENSION AND STRAIN

Strain and tension forces occur due to mechanical stretching or alterations in the ECM creating tensile forces. Substrate stiffness and substrate anchorage is an important factor in the generation of tensile forces and can directly impact cancer cell response. Despite accumulated knowledge of the pro-metastatic effects of strain in widely studied cancers, such as breast cancer, the mechanisms by which strain is “felt” and responded to by cancer cells, and other cancer related cells, remains incompletely understood.^69,70^ Additionally, there are two distinct modes of strain experienced by cancer cells that can contribute to cancer cell progression: (i) ECM stiffness-based tensile strain and (ii) strain caused by outside forces. ECM stiffness-based strain is caused directly by the stiffening of the ECM, often experienced by growing tumors. Pathological ECM stiffness leads to increased tensile forces in the cytoskeleton.^71,72^ The second type of strain occurs simply due to biological phenomena, such as mechanical strain from peristalsis in the colon and uterus.^47,73^ The complexity of the interaction between tensile forces and a tumor requires intentional models to facilitate *in vitro* study.

Inherently, the varying and complex nature of ECM stiffness-based strain makes standard, stiff plastic tissue culture dishes inadequate in studying the importance of tensile forces and ECM stiffness. These models are not tunable or mimetic of the stiffness and modulus of *in vivo* tissues where cancer cells reside. Therefore, researchers have created 2D and 3D model systems that better exhibit the characteristics of tensile forces and ECM interactions *in vitro*. For example, Kopanska *et al.* utilized 3D CT26 tumor spheroids embedded in a biomimetic collagen I matrix to study how tensile forces within the ECM dictate invasion. In this model, researchers show that cellular contractile forces immediately deform the ECM leading to tensile radial forces within the surrounding matrix. The resulting tension causes an increase in invasion. In fact, when the collagen gel was cut to reduce the tensile strain, less invasion was observed from the colorectal spheroids. By evaluating these high and low tensile states of the ECM, researchers determined that there is a direct link between tensile forces in the ECM and invasion. Ultimately, they presented a tension-based model that contradicted previous models relating invasion to the elastic modulus.^74^ These results suggest that tensile force created by the ECM may be a key factor in cancer cell invasion and the subsequent metastatic cascade. Stiff and soft materials employed in the study of tensile strain have been well reviewed in a cancer context.^75,76^ In an additional model of tensile forces in the TME, a 3D tension bioreactor capable of minute specifications of collagen hydrogel stiffness was designed by Cassereau *et al.* This work was aimed at investigating the interaction between ECM stiffness leading to tensile forces and mammary tumor phenotype (Fig. 2).^77^ By mechanically loading collagen hydrogels covalently conjugated to a polydimethylsiloxane membrane, they induced hydrogel stiffening at varying levels of intensity (0.4–4 kPa). This tunable model demonstrated that ECM stiffness independently enhanced mammary organoid tumor cell invasion. By eliminating variables, such as composition and pore size, this novel model isolated the effect of tensile forces on tumor cell invasion. Using microbial transglutaminase-based variations in gelatin hydrogels, Woods *et al.* demonstrated that stromal cells like mammary fibroblasts also respond to changes in ECM compliance by demonstrating activation into a myofibroblast state, which can further breast cancer metastasis.^78^ The continued study at this intersection has brought about a promising therapeutic strategy for resolving tensile strain-induced complications and malignancy in tumors: the specific targeting of ECM stiffness.

Strain caused by biological phenomena, excluding matrix stiffness caused by a tumor, is distinct from ECM stiffness-based strain. This type of strain occurs in the case of mechanical stretching or multi-axial strain from organ systems. Specifically, uniaxial tensile strain is experienced by skeletal muscle and osteoblasts,^79,80^ and multiaxial strain is experienced by colon cells, endometrial cells, and blood cells.^81–83^ A unique, tunable bioreactor was designed to mimic the biaxial strain present in the colon, vasculature, and other organ systems.^84^ It may be noted that strain in the colon, among other systems, is cyclic strain, or based on repeated fluctuations of force. Another model of colorectal cancer utilized organ-on-a-chip technology to concurrently study the effects of cyclic peristaltic forces (shear stress and strain). This investigation determined that invasion was substantially increased in both monolayer and organoid cultures in the mechanically stimulated condition compared to static controls.^41^ In this novel colorectal cancer organ-on-a-chip model, invasion, evidenced by movement from an epithelial to endothelial compartment, mimicked intravasation that is found in the *in vivo* setting. Cyclic strain is continuously present in the colon, and Strelez *et al.* successfully created an on-chip model to study these forces *in vitro*. Similarly, a hydraulic force cell culture chip model with a deformable substrate was used to study the effects of cyclic tensile stress on the ability of myofibroblasts to dictate lung cancer cell migration.^85^ In this model, cyclic tensile strain was applied to myofibroblasts resulting in diminished migration of cancer cells with a reduction in alpha smooth muscle actin. In this case, the effect of cyclic tensile strain secondarily impacted cancer cell response. Similarly, FlexCell strain units are widely applied to explore the effects of mechanical stretch and strain *in vitro*.^86,87^ In one example, Wang *et al.* evaluated breast cancer cell response to oscillatory strain consistent of that found in the *in vivo* breast tumor microenvironment. Using the FlexCell tension system, researchers determined that oscillatory strain promoted monolayer breast cancer cell proliferation and migration, both of which are contributing factors to metastatic potential.^86^ Another application of the FlexCell system is in the study of ovarian cancer cell response to tension forces by Martinez *et al.*^88^ The impact of mechanical tensile forces on the progression and metastasis of ovarian cancer is minimally understood so this work sought to explore this connection. Using applied oscillatory tension for 3 days, SKOV-3 and OVCAR-8 cells demonstrated enhanced cell proliferation and increased metastatic phenotypes via increased transwell migration and invasion. With the use of a tension-based model system, this study found that mechanical forces are influential in ovarian cancer progression and metastasis. These examples defend the notion that models that employ other cells, or 3D systems to begin with, have a better advantage at studying cancer metastasis rather than 2D monolayer culture.^89–92^ Even the initiation of metastasis *in vivo* involves significant orchestration between several different cell types in the tumor microenvironment better recapitulated by 3D and multi-cell systems.

Each of these strain-based systems work to incorporate aspects of biological strain to determine cancer cell response. Future studies on the interactions between strain and cancer malignancy should further investigate the connection between invasive potential and tensile strain caused by organ function and ECM contractions, when possible, through 3D culture. The incorporation of multiple physiologically relevant sources of strain, through the integration of bioreactor, organ-on-a-chip, ECM-focused models, and other emerging strategies, is vital to understanding the relationship between strain and cancer malignancy.

## IV. BIOENGINEERING STRATEGIES TO STUDY CANCER CELL RESPONSE TO SOLID STRESS AND COMPRESSION

In the TME, components of solid stress and compression occur and alter cell response. As tumors continue to grow in size, interior compression drives inner cells to see a decrease in proliferation, but a concurrent decrease in apoptosis.^93^ In addition, with tumor microenvironment compression, unregulated tumor growth can result in elevated interstitial pressure, thereby increasing the compressive forces on the tumor as a whole.^28^ Specifically, solid stress and compression drive increases in EMT genes and migratory capabilities in breast and ovarian cancer cells.^94–96^ Solid stress and compression are, therefore, best recapitulated in a 3D model to account for the multi-axial forces felt by the cancer cells.

Current *in vitro* compression models vary from biomaterial- to bioreactor- to microscale-based platforms to apply compression on cancer cells in a localized and dynamic manner. In one example by Tse *et al.*, a piston filled with adjustable weight applied a constant force to an agarose disk in contact with a monolayer of mammary tumor cells on a transwell membrane.^94^ This work primarily evaluated cell migration under constant loading force, such as that experienced in the mammary duct due to uncontrolled proliferation. In this model, researchers demonstrated that compressive stress stimulated the migratory capability of mammary carcinoma cells. This method provided initial insight into bulk, static compression in a cancerous setting. A similar study by Klymenko *et al.* evaluated ascites-induced compression on ovarian cancer multicellular aggregates (Table I). By employing the use of a Flexcell compression system, researchers applied compression to ovarian cancer multicellular aggregates similar to the compressive force created by intraperitoneal pressure in the ascites (Fig. 2). Aggregates were fully encapsulated in a porous hydrogel and compressed for 6 or 24 h. Interestingly, in short term compression, expression was decreased whereas in long term compression, expression was enhanced in genes related to epithelial to mesenchymal transition. Potentially, in the extended compression condition, cells resorted to more mesenchymal like expression to become more motile and escape the aggregate formation to relieve pressure. These results were observed in both the E-cadherin and N-cadherin-expressing cell types tested. Sustained compression created by intraperitoneal fluid pressure, therefore, might contribute to the generation of more metastatic phenotypes in multicellular aggregates. In another larger scale study by Novak *et al.*, a 3D bioreactor was used to study ovarian cancer cells and the effects of hydrostatic pressure leading to compression (Fig. 2).^97^ This work relied on the use of a unique compression bioreactor that distributes cyclic or static pressure within a cell laden interpenetrating hydrogel. The pressure distribution resulted in compressive forces in the range of 3.9–6.5 kPa experienced by the ovarian cancer cells. Results from this work indicated that both cyclic and static compression induced invasive morphology, proliferation, and chemoresistance of high grade serous ovarian cancer cells via CDC42. Compression also plays a role in glioblastoma where fluid accumulation compresses the tumor and surrounding normal tissue resulting in decreased drug uptake. One model by Ilkhanizadeh *et al.* examined the impact of compression on glioblastoma using a custom-built compression bioreactor.^98^ Glioblastoma tumor spheres were embedded in 3D hyaluronic acid hydrogels and exposed to compression for 48 h. This work ultimately resulted in the discovery of an antisecretory factor that can be targeted for antitumor activity by reducing interstitial fluid pressure. These examples all demonstrate model systems that incorporate compression forces that mimic various physiological events with the common goal of determining how compression impacts cancer cells.

The use of small-scale engineering can also be employed to mimic the physiological environment of tumors *in vitro*. In a microdevice-based study by Onal *et al.*, researchers created a micro piston platform out of a polydimethylsiloxane (PDMS) membrane. In an effort to better mimic physiologically relevant compression found in human tumors, this model allowed for precise control of a micro piston to create dynamic and controlled compressive stimulation by modulating the amount and duration of the applied pressure, shape and size of the actuator, and its planar localization in the microchannel.^99^ Following cyclic and varying compression profiles applied to SKOV-3 ovarian cancer cells, researchers evaluated nuclear and actin deformation patterns. Further work with this model will encompass additional cell types and extended downstream analysis to better understand compression's role in the peritoneal cavity in the metastatic spread of ovarian cancer. Compression and solid stress forces impact cancer cells uniquely in numerous cancer types, as evidenced by the models outlined above. Future work should continue to incorporate additional characteristics of the TME in compression studies to better understand the complex nature of these forces interacting with the ECM, cancer cells, and other native cell types.

## V. CONCLUSIONS AND FUTURE PERSPECTIVES: MECHANICAL FORCES IN CONJUNCTION

Mechanical forces in the tumor microenvironment, and throughout the metastatic cascade, do not influence cancer malignancy on isolated single scales. Rather, tumors and circulating tumor cells are subjected to multiple forces simultaneously or in succession, at multiple scales (sub-cellular all the way to tissue scales) and their overall response to mechanical stimuli may depend on the combination or order of force application. Additionally, the downstream effects of a biomechanical force can affect another force, with corresponding impacts on a tumor. For example, tension present at cell–cell junctions due to cellular adhesion connecting the contractile cytoskeletons of adjacent cells can cause leakage of fluids from within the tumor.^106^ This effect compounded with a pressure gradient caused by interstitial fluid^57–59^ can increase chemoresistance and metastasis.^107^ Similarly, the solid stress caused by tumor growth can compress blood vessels,^108^ thereby reducing perfusion and promoting angiogenesis.^109^ Increased angiogenesis in turn contributes to tumor growth, creating a positive feedback loop. Complications due to multiple force-induced effects on cancer malignancy require innovative models of combinatory study. The necessary complexity of models capable of mimicking multiple physiological forces can present obstacles to experimental study, suggesting the application of computational models. However, computational methods, which have been thoroughly reviewed in this context,^110^ present their own concerns. Specifically, there is an inherent limitation of physiological factors outside of those mathematically understood and modeled. *In vitro* studies using physiological models remain extremely important to understanding the interplay between multiple forces and malignancy.

All *in vitro* systems inherently have multiple forces present, just not always as tightly controlled variables. For example, all experimental substrates have some relative stiffness values and any time media is changed or a plate is moved there is the element of shear stress. Mechanical forces have always been a source of variation in *in vitro* studies, but current models work to incorporate specific, physiologically relevant forces as a true experimental variable. To that end, researchers have designed bioreactors with multiple forces involved to study the effects of combined physiological forces.^111,112^ One bioreactor, created by Clevenger *et al.*, uses a rotating screw drive and peristaltic pump to produce multi-axial strain and fluid shear stress levels that can be tuned to mimic multiple organ systems (Fig. 2).^84^ By seeding the bioreactor with monolayer colorectal cancer cells and tuning the mechanical components to mimic the forces present in the colon, they determined that HCT116 colorectal cancer cells demonstrate increased metrics of progression in response to peristalsis forces. This model was unique for the study of peristalsis in its incorporation of both fluid shear stress and multi-axial strain and can be tuned to study additional organ systems where peristalsis is present. Similarly, Meza *et al.* developed a concurrent cone-and-plate shearing and membrane suction stretching device to study endothelial cell response to these mechanical stimuli.^113^ This system can be easily repurposed to investigate concurrent forces and cancer malignancy. While few *in vitro* simultaneous force models have been designed and employed in this context, they provide physiological relevance unreachable by single force models. Future studies should attempt to incorporate multiple accurately recreated biomechanical forces to increase the understanding of the impact of all the forces present in the TME.

Another mostly unexplored but important aspect of the interaction between mechanical forces in the body and cancer malignancy is the contribution of the fourth dimension: time. Strain in the colon, for example, is greatly influenced by the circadian rhythm,^114^ resulting in fluctuation of the force exerted on a colorectal cancer cell. Additionally, the circadian rhythm of the heart causes variation in fluid flow velocity throughout the circulatory system which could have a direct impact on cancer cell dissemination.^115^ While there has been increased investigation recently into the relationship between the circadian rhythm and cancer metastasis,^116,117^ the link is not completely understood. Similarly, the variation of mechanical force intensity throughout the body and how it may affect malignancy has recently been explored.^42,117^ Mechanical memory, or the continued response to past mechanical environments, of both cancer and cancer-related cells is also an important emerging area of study.^118–120^ The idea that cells can commit to memory a past mechanical stimulus and continue to alter their response is fascinating. Importantly though, we are often so focused on functional phenotypes, that we sometimes ignore the broader sense of what these forces can do at the molecular or genetic and epigenetic level to induce mechanical memory. To this end, multidisciplinary approaches that reach across the aisle to cell and molecular biologists and bioinformaticists must take place to unravel the molecular complexity of mechanobiology.^121^ The numerous models and materials discussed in this review are relevant systems to further explore the concept of mechanical memory and would be greatly strengthened in conjunction with advanced cell biology and bioinformatics insight.

Another avenue of importance in the study of time's role in mechanics and cancer is in dynamic materials that allow for the modeling and control of degradability and viscoelasticity over time.^76,122^ Additionally, the incorporation of mechanoresponsive materials, such as hydrogels, would allow for the exploration of the effects of dynamic mechanical stimuli on cells.^123^ Continued work focused on exploring these important material characteristics will further contribute to the understanding of mechanical forces and cancer malignancy with respect to time. Importantly, however, the interplay of time, biomechanical forces, and cancer metastasis is yet to be explored and would be an interesting avenue for future work in this field.

In this review, we discussed the many ways current model systems can be used to analyze the effects of physical forces on cancer cell progression and metastasis. Shear stress, tension, and solid stress all have an impact on a cancer cell's ability to metastasize, but the major question is how exactly these forces influence cell metastasis. Importantly, advancing technologies are allowing new ways to study these cellular processes in response to mechanical forces. By working to address this question, we can better understand how to detect, treat, and prevent cancer progression and metastasis.

## Data Availability

Data sharing is not applicable to this article as no new data were created or analyzed in this study.
